# A Detailed Evaluation of the Suitability of Waterbird Habitats in Hangzhou Bay National Wetland Park—A Differential Analysis of Wading Birds and Swimming Birds

**DOI:** 10.3390/biology15181617

**Published:** 2026-09-14

**Authors:** Zixin Fan, Shengwu Jiao, Xuexin Shao, Ming Wu, Long Zhang, Xiajuan Xu, Xingna Lin

**Affiliations:** 1 Wetland Ecosystem Research Station of Hangzhou Bay, Research Institute of Subtropical Forestry, Chinese Academy of Forestry, Hangzhou 311400, China; 2 State Key Laboratory of Wetland Conservation and Restoration, Beijing 100091, China; 3 Zhejiang Provincial Key Laboratory of Wetland Intelligent Monitoring and Ecological Restoration, Hangzhou 311121, China; 4 College of Forestry, Nanjing Forestry University, Nanjing 210037, China

**Keywords:** waterbirds, Hangzhou Bay, habitat suitability, habitat suitability index model, fine-scale assessment, wading birds, swimming birds

## Abstract

Hangzhou Bay is an estuarine ecosystem on the Yangtze River Delta and a key wintering habitat for waterbirds. However, suitable waterbird habitats have been degrading and fracturing. Therefore, we aimed to assess waterbird habitat suitability and identify the principal factors affecting quality in Hangzhou Bay National Wetland Park. Habitat suitability index (HSI) models were constructed for wading and swimming birds using water source conditions, disturbance levels, concealment, and food abundance. The models adopted consistent factor weights but differentiated the grading criteria for the waterbird groups. Applicability was validated using bird survey data collected in 2025, and habitat suitability was quantitatively assessed using field and literature data. Food abundance and water source conditions were dominant factors influencing habitat suitability; concealment and disturbance levels had minor effects. The abundances of wading and swimming birds increased with HSI values, although limited validation sites constrained these relationships. Positive trends indicate that the HSI models may capture the spatial variation in habitat use. Grade III or higher areas comprised 96.67% and 74.21% of the total area for wading and swimming birds, respectively; no Grade I habitats were identified. Overall, the study area provided favorable habitats for waterbirds, although habitat suitability differed between the groups. These findings may provide a quantitative reference for the targeted conservation and restoration of waterbird habitats in the Hangzhou Bay wetland.

## Simple Summary

Coastal wetlands provide essential feeding and resting habitats for waterbirds; however, these habitats are increasingly affected by human activities, altered water conditions, and vegetation succession. This study assessed habitat suitability for wading and swimming birds in the Education and Exhibition Zone of Hangzhou Bay National Wetland Park. As these two groups differ in their habitat requirements, separate suitability criteria were developed based on water source conditions, human disturbance, concealment, and food abundance. Bird survey data collected in 2025 were used to evaluate whether the assessment results reflected the number of birds recorded at the six survey sites. Food abundance and water source conditions are the most important factors influencing habitat suitability. The sites with higher habitat suitability scores supported more birds; however, this relationship was stronger for wading than for swimming. Habitats of moderate suitability or higher accounted for 96.67% of the study area for wading birds and 74.21% for swimming birds, and no unsuitable habitats were identified for either group. However, the spatial distribution of suitable habitats differed between the two groups. These findings can help wetland managers identify important habitat areas and develop targeted conservation and restoration measures for different groups of waterbirds.

## 1. Introduction

Wetlands are vital habitats for waterbirds and are essential for their survival, reproduction, and migration. Habitat quality directly affects waterbird distribution and population dynamics. The habitat suitability index (HSI) can quantitatively assess the suitability of different areas for the survival and activity of target species by comprehensively analyzing the relationship between species distribution and environmental factors. This index can also be used to predict trends in habitat change, thus providing technical support for species monitoring, habitat identification, and ecological conservation planning [1,2]. Assessing the suitability of waterbird habitats and identifying their key limiting factors are important for current wetland conservation management and precise restoration.

Assessment methods for waterbird habitat suitability include maximum entropy, ecological niche factor analysis, logistic regression, and HSI model [3,4,5]. The maximum entropy model has a high prediction accuracy for waterbird habitat suitability, but it requires high-quality and high-quantity input data [6]. The ecological niche factor analysis model requires only waterbird distribution data during the assessment process; however, it has limited capacity to represent ecological processes affecting waterbird habitat suitability [7]. The logistic regression model is computationally simple, easy to construct, and straightforward to apply; however, it requires simultaneous access to data on species presence and absence, which are usually difficult to obtain in field surveys [8]. The HSI model can be used to evaluate habitat quality, identify potentially suitable habitats, and be applied to wetland waterbird studies targeting habitat characteristic analysis and spatial optimization [9]. However, conventional HSI models were constructed separately for each species. The evaluation indicators, factor weights, and suitability functions of different species models may change simultaneously, resulting in the lack of a unified comparison benchmark for evaluation results across different species or ecological functional groups [10,11]. Beyond this comparability issue, no existing approach has accommodated multiple functional guilds simultaneously within a unified framework, while preserving the ecological distinctiveness of each group.

To address this gap and resolve the tradeoff between comparability and ecological specificity, we developed a differentiated HSI framework that retains common evaluation indicators and factor weights for both wading and swimming birds, enabling a guild-level, landscape-scale evaluation of habitat suitability that provides spatially explicit information for integrated wetland restoration and conservation planning. This design is ecologically supported by the major parameters of waterbird habitat quality, such as hydrological conditions, vegetation structure, food availability, and human disturbance, being shared across guilds, although their preferred ranges and response patterns may vary among groups [12,13]. Waterbirds of different ecological functional groups exhibit differences in their responses to identical habitat factors and suitability thresholds, owing to variations in their morphological characteristics, feeding modes, and resource-utilization strategies [14,15,16,17]. Wading birds are mainly distributed in shallow water areas; their foraging habitats are mostly open bare tidal flats with unobstructed views [18,19]; their diets are dominated by animal resources such as fish, shrimp, and benthic organisms [20,21,22]; and their breeding areas are primarily located at the edge of woodlands [23]. In contrast, swimming birds are mainly distributed in deep-water areas; both their foraging and breeding activities occur in areas with medium to high vegetation coverage, and their diets are mainly composed of plant resources such as rhizomes [22,24]. Given these distinct habitat preferences, we retained the common factor weights for both guilds to reflect landscape-level environmental supply, while applying group-specific grading criteria to capture their divergent habitat requirements.

Located on the south bank of Hangzhou Bay, Hangzhou Bay National Wetland Park is a critical stopover site in the East Asian–Australasian Flyway. The area supports a rich assemblage of waterbird species, with wading and swimming birds being the dominant groups. It features diverse coexisting habitats, such as tidal flats, shallow waters, and reed marshes, with high landscape heterogeneity, making it an ideal study area for conducting refined waterbird habitat suitability assessments. Taking the Public Education and Exhibition Zone of Hangzhou Bay National Wetland Park as the study area, four influencing factors were selected: water source condition, shelter degree, disturbance condition, and food abundance. Combined with the expert scoring method and analytic hierarchy process [25], based on the main utilization patterns of waterbirds in wetland spaces, the waterbirds in the study area were operationally classified into two functional groups: wading and swimming birds. HSI models with common factor weights and group-specific grading criteria were constructed for the two groups. Bird survey data from 2025 were used to evaluate the applicability of the HSI models. Furthermore, the models were applied to carry out refined evaluations of the habitat suitability of swimming and wading birds in the study area to provide a scientific basis for conservation management and ecological restoration of bird habitats in the wetland.

## 2. Materials and Methods

### 2.1. Overview of the Study Area

**Figure 1:**
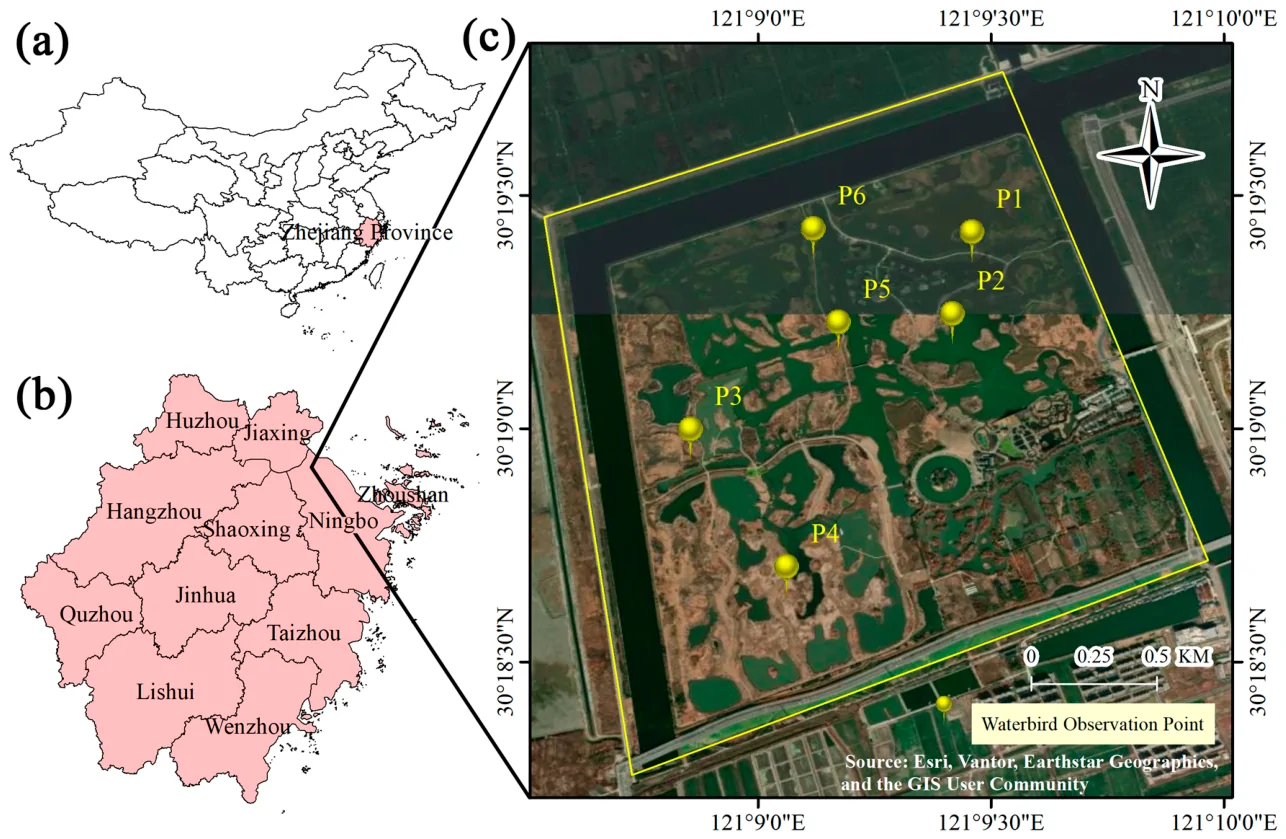
Study area and waterbird survey sites. (**a**) Zhejiang Province in China; (**b**) study area within Zhejiang Province; and (**c**) boundary of study area, delineated by yellow polygon, and six waterbird survey sites (P1–P6).

Hangzhou Bay National Wetland Park (121°3′28.63″ E–121°9′54.89″ E, 30°17′1.31″ N–30°23′54.77″ N) is on the south bank of Hangzhou Bay, with a total area of 6376.69 ha. This region is one of the most abundant for wintering waterbirds along the eastern Chinese mainland coast and a critical stopover site on the East Asian–Australasian Flyway. This region acts as a wintering ground and migratory stopover for globally endangered species, including the Saunders’s gull, Black-faced Spoonbill, and Dalmatian pelican, boasting extremely significant ecological significance [26,27]. This area falls in the subtropical monsoon climate zone, with a multi-year average temperature of 16.0 °C, annual average precipitation of 1486.4 mm, and annual sunshine duration of 2035.3 h. Based on the conservation and utilization objectives, the park is divided into several functional zones for ecological conservation (3150.28 ha), restoration and reconstruction (1962.57 ha), education and exhibition (379.18 ha), rational utilization (853.13 ha), and management and service (31.55 ha). In this study, the education and exhibition zone was selected as the study area. Located within the core of the Wetland Park, this zone covers a total area of approximately 380 ha (Figure 1). The study area features high habitat heterogeneity, providing diverse habitats for waterbirds with different ecological habits.

### 2.2. Habitat Suitability Model Construction

#### 2.2.1. Construction of Evaluation System and Factor Classification

The evaluation factors were organized into four hierarchical levels: goals, criteria, indicators, and alternatives. Based on the current conditions of waterbird habitats, previous studies, and expert consultations, a waterbird habitat suitability assessment was defined as the goal layer. The criterion layer comprised four factor categories: water conditions, concealment, disturbance condition, and food abundance [13,28]. In the indicator layer, specific evaluation factors capable of characterizing the aforementioned ecological conditions were selected (see Supplementary Table S1 for details). The evaluation factors were selected based on their ecological relevance, data availability, spatial continuity, and temporal correspondence with the waterbird survey period. Although factors such as seasonal hydrological and water quality conditions and substrate characteristics may influence waterbird habitat selection, they were not directly incorporated into the evaluation system because of limitations in data coverage and spatiotemporal resolution at the scale of the study area. In the alternative layer, the differences among waterbird groups in their responses to habitat factors were considered. Based on their principal activity and foraging habitats as well as their predominant locomotion and foraging modes, waterbirds were operationally classified into two functional groups: wading and swimming birds [22,29]. Based on the differing habitat requirements of these two groups, the influencing factors within the study area were classified into graded numerical ranges, resulting in a five-level classification system for the factors affecting waterbird habitats (see Supplementary Tables S1 and S2 for details).

#### 2.2.2. Weight of Evaluation Factors

In this study, we used the same factor weights for both wading and swimming birds as they reflect the relative contributions of different environmental dimensions to overall habitat suitability at the landscape scale. This is an objective property of the study area rather than a guild-specific attribute. An analytic hierarchy process (AHP) was used to determine the weights of the evaluation indicators.

The expert consultation questionnaire was designed based on a preliminary literature review, actual environmental characteristics of the study area, and hierarchical structure of the evaluation system. The questionnaire mainly included pairwise comparisons among evaluation factors at the criterion and indicator levels (see the separate Supplementary File “Questionnaire” for details). We invited 15 experts in wetland ecology, ornithology, and related fields to complete pairwise comparison questionnaires based on Saaty’s 1–9 scale (Supplementary Table S3), with each expert assigned equal weights in the group decision process. The 15 experts were selected based on the following criteria: (1) holding a doctoral degree in a relevant field or having been engaged in ornithological research for at least five years; (2) familiarity with waterbirds and their habitats in the study area or in similar wetlands; and (3) coming from different institutions. These criteria were intended to ensure professionalism and representativeness of the expert panel. The final panel comprised 15 experts from five different universities and research institutes with expertise in waterbird ecology, wetland ecology, habitat assessment, and related environmental sciences. An individual-judgment matrix was constructed for each expert. The consistency of each matrix was evaluated using the consistency ratio (CR). A matrix was considered acceptable when the CR was <0.10. Matrices that did not meet this criterion were returned to the experts for revision and retesting, until they passed the consistency test. The consistency index (CI) and CR were calculated to evaluate the consistency of the judgment matrices. The calculation formula is as follows:

(1) $$\mathrm{CI}=\frac{\lambda \max-n}{n-1}$$

where *λ*max is the maximum eigenvalue of the judgment matrix; *n* is the order of the judgment matrix (i.e., the number of factors).

(2) $$\mathrm{CR}=\frac{\mathrm{CI}}{\mathrm{RI}}$$

where RI is the random consistency index.

An integrated judgment matrix was constructed using the matrices that were derived from each expert’s pairwise comparisons, passed the consistency test, and were aggregated using the geometric mean method [30,31]. This integrated judgment matrix was subsequently subjected to an additional consistency test. The yaahp software (version 10.1) was used to calculate the local weights of indicators at each hierarchical level, and hierarchical synthesis was performed to obtain the overall weights of each evaluation factor relative to the goal layer [30].

To maintain a consistent evaluation, the same environmental factor weights were applied to both the wading and swimming birds, with the two functional groups treated as parallel evaluation objects [13]. Differences in habitat requirements are reflected in group-specific grading criteria and suitability thresholds for individual indicators [15,25].

#### 2.2.3. Habitat Suitability Assessment

The HSI was calculated based on the classification and assignment of the evaluation factors using the following formula:

(3) $$\mathrm{HSI}=\sum_{i=1}^{n}S_{i}W_{i}$$

where *n* is the number of evaluation factors, *Si* is the suitability grade of the *i*th evaluation factor, and *Wi* is the habitat suitability weight of the *i*th evaluation factor [28].

### 2.3. Data Sources

High-resolution remote-sensing imagery with a spatial resolution of 0.5 m, acquired in April 2025 from Google Earth Pro (version 7.3.7.1327, 64-bit), was used as the primary data source. Land use types within the study area were extracted through visual interpretation, and the results were verified and corrected using field surveys. Land-use types were classified into eight categories: buildings, artificial vegetation, grassland, forest land, shrubland, tidal flats, water bodies, and hardscapes.

**Figure 2:**
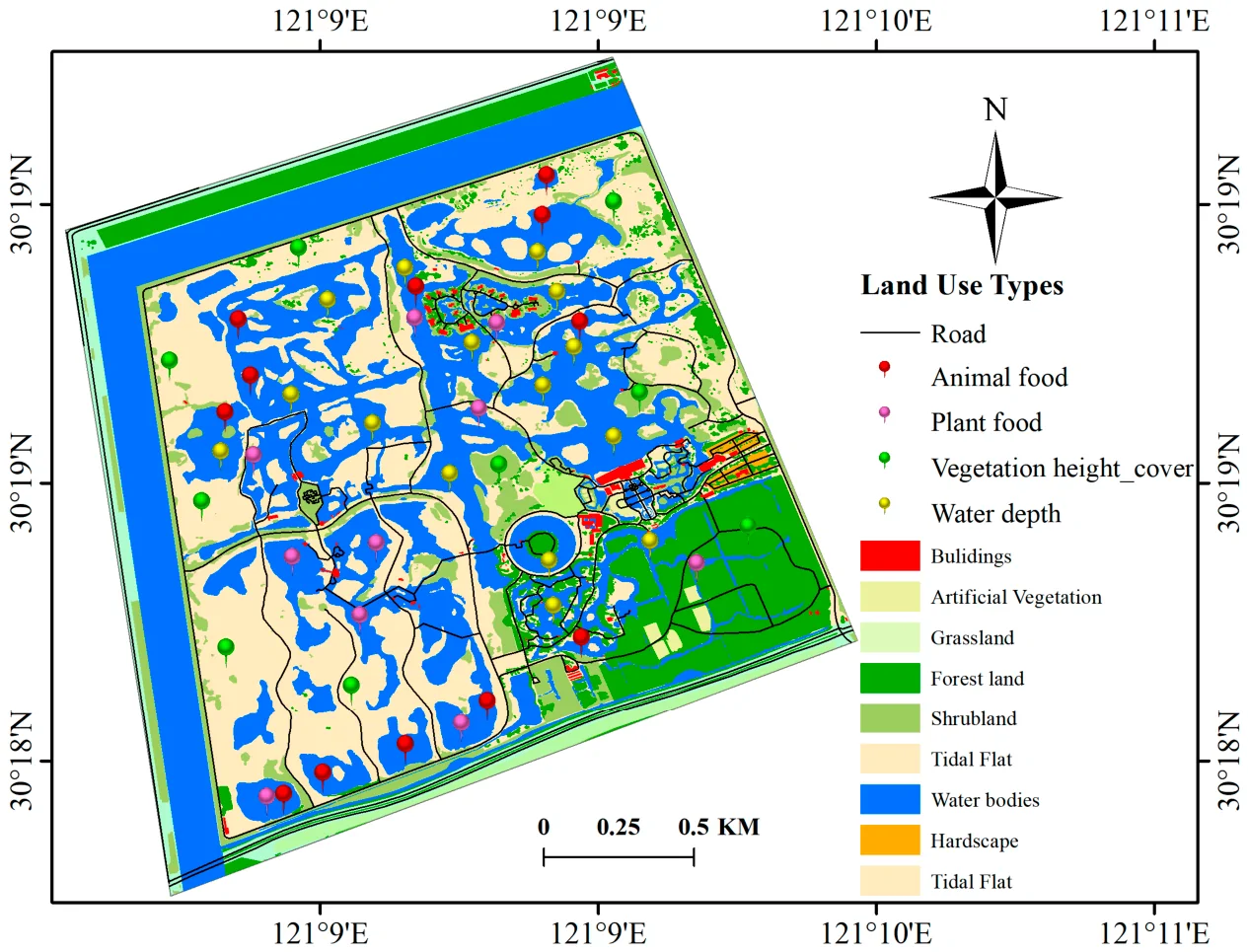
Spatial distribution of land-use types and field survey sites in the study area. Red points represent animal food sampling sites, purple points represent plant food survey sites, green points represent vegetation height and vegetation cover survey plots, and yellow points represent water-depth survey sites.

Based on the land-use distribution map and the relevant spatial information obtained from field surveys, spatial analysis in ArcMap 10.8 was used to generate distribution maps of the waterbody area derived through visual interpretation of remote-sensing imagery, patch number quantified using a moving-window neighborhood analysis of the land-use raster, and distances to water sources, roads, and visitor stopping points calculated using Euclidean distance analysis within the study area. The slope data for the study area were derived from topographic maps provided by local surveying and mapping authorities. Water depth was measured in situ across most water areas using an ultrasonic depth sounder (YP-CS150, Shandong Youyunpu Optoelectronics Technology Co., Ltd., Weifang, Shandong, China), whereas depths in areas where field measurements were not feasible were estimated based on the difference between the water surface elevation and the corresponding elevation data. Representative vegetation plots were selected based on the vegetation types identified during the field surveys, and vegetation height and cover were measured within the same quadrats. The mean vegetation height obtained for each vegetation type was assigned to the corresponding areas. The locations of the field surveys for water depth, vegetation height, and vegetation cover are shown in Figure 2.

Plant food abundance was assessed based on the spatial distribution of the aquatic vegetation available to waterbirds. Field surveys were conducted in the summer to identify the distribution ranges of edible aquatic vegetation within the study area. The boundaries of the aquatic vegetation patches were recorded in the field using a handheld GPS receiver (Garmin eTrex 22x, Garmin International, Inc., Olathe, KS, USA). The surveyed vegetation patches were further corrected and digitized in ArcGIS 10.8 using high-resolution Google Earth imagery. The digitized aquatic vegetation data were converted into raster layers, and focal statistics were applied to calculate the proportion of aquatic vegetation within a 25 m radius circular neighborhood around each raster cell, representing the local availability of plant food resources. The survey locations are shown in Figure 2.

Animal food abundance was determined based on trapnet sampling data. The sampling was conducted once per season (spring, summer, autumn, and winter) with three trap nets of the same specifications (6.4 m in length, 2.0 cm mesh size, 48 cm in height, and 60 cm in width) deployed at each sampling site for 10–15 consecutive days. The captured animals were identified, and their species, number, body mass, and body length were recorded before being released at the capture sites. The biomass captured by each trap net was used to represent the relative abundance of animal food resources, expressed as g·trap^−1^. Seasonal biomass data were spatially interpolated using the inverse distance weighting (IDW) method to generate continuous distribution layers of animal food abundance for the subsequent habitat suitability assessments. The locations of the animal food sampling sites are shown in Figure 2.

Based on the aforementioned data sources and processing procedures, the HSI assessment in this study was primarily used to compare the overall annual spatial patterns of habitat suitability and the differences between wading and swimming birds, rather than to characterize monthly changes in habitat conditions. Therefore, the annual HSI assessment was consistent with the comparative objective of this study and provided a common spatial basis for comparing habitat suitability between the two waterbird functional groups.

### 2.4. Waterbird Survey Methods

From January to December 2025, monthly waterbird surveys were conducted in the study area using the point-count method. Six fixed observation points were set up, covering major habitat types, such as open water, shallow tidal flats, reed marshes, and artificial stopover sites (Figure 1). After arriving at the observation point, the observer waited for 5 min to reduce disturbance. A 20× monocular telescope (Carl Zeiss Victory DiaScope 85 T FL, Carl Zeiss Sports Optics GmbH, Carl-Zeiss-Straße 22, 73447 Oberkochen, Germany) and 8× binoculars (ZEISS Terra ED 42, Carl Zeiss Sports Optics GmbH, Carl-Zeiss-Straße 22, 73447 Oberkochen, Germany) supplemented with digital camera photography were used to record the species and quantity of waterbirds within the field of view. Each observation lasted for no less than 15 min, with an effective observation distance of 200 m.

### 2.5. Validation of Habitat Suitability Model

To verify the applicability of the HSI model to the study area, the observed counts of swimming and wading birds within the effective observation radius were compared with the average HSI values of the corresponding areas. The average HSI value within the effective observation distance of each survey point was calculated as follows. First, the pixel average values of all evaluation factor rasters within the effective observation distance of each survey point were extracted, and weighted summation was performed according to Equation (3) and the weights in Table 1. Spearman’s rank correlation was used to examine the relationship between the HSI values at the survey sites and cumulative bird counts, as this rank-based method does not require data to be normally distributed. Both annual and monthly data were used to assess the spatial and temporal correspondence between the HSI and bird abundance. A leave-one-site-out (LOO) sensitivity analysis was performed to evaluate the influence of the individual sites [32]. Based on established HSI validation practices, we considered the model acceptable when the mean predicted HSI was significantly positively correlated with mean observed bird abundance (*p* < 0.05) and Spearman’s ρ ≥ 0.30, and when this positive correlation remained directionally consistent across the LOO analyses [33,34,35].

### 2.6. Habitat Suitability Calculation and Spatial Distribution

Using the validated HSI model, habitat suitability was calculated for each grid in the study area. The HSI value of each grid was calculated pixel-by-pixel according to Equation (3), and the results were divided into five grades using the equal interval classification method: highly suitable (V), good suitability (IV), moderately suitable (III), low suitability (II), and unsuitable (I). In accordance with the classification criteria of the HSI (see Supplementary Table S4 for details), spatial distribution maps of habitat suitability for wading and swimming birds were generated, and the area and proportion of each grade of habitat were statistically analyzed.

## 3. Results

### 3.1. Waterbird Species Composition and Richness

In 2025, 50 waterbird species belonging to 13 families and 7 orders were recorded at the six observation sites (see Supplementary Table S5 for details). Twenty species belonged to Charadriiformes, and 11 each to Pelecaniformes and Anseriformes, accounting for 40% and 22% of the total waterbird species recorded in the study area, respectively. Several protected species were recorded, including the Black-faced Spoonbill, which is listed as a Class I National Key Protected Wild Animal in China, and the Eurasian Spoonbill, Mandarin Duck, Greylag Goose, and Pheasant-tailed Jacana, which are listed as Class II National Key Protected Wild Animals. According to the IUCN Red List of Threatened Species, the Black-faced Spoonbill is globally endangered, the Eurasian Spoonbill is globally vulnerable, and the falcated duck and black-tailed godwit are globally threatened species [28].

The recorded species were classified as either wading or swimming birds. The following approaches have been used in previous waterbird functional group studies to classify feeding modes and feeding habitats [29,36,37]: (1) predominant locomotion and foraging mode. Species that maintained foot contact with the substrate and primarily moved and foraged by standing, walking, or wading were classified as wading birds. Those that primarily relied on buoyancy and moved or foraged by floating, swimming, upending, or diving were classified as swimming birds. (2) Predominant foraging habitat and its spatial relationship with the water–substrate interface. Species that mainly used shallow-water areas, mudflats, shorelines, and aquatic–terrestrial transition zones, with the water–substrate interface serving as their principal foraging space, were classified as wading birds. Species that mainly used continuous open-water surfaces and aquatic environments, with the water surface, upper water layer, or water column serving as their principal activity and foraging space, were classified as swimming birds. This classification represents a first-order functional grouping based on wetland habitat-use characteristics and is intended primarily to reflect broad differences in foraging modes and foraging habitats among waterbirds. Further differentiation in diet and microhabitat use may still occur within each functional group.

### 3.2. Abundance and Spatial Distribution of Waterbirds

**Figure 3:**
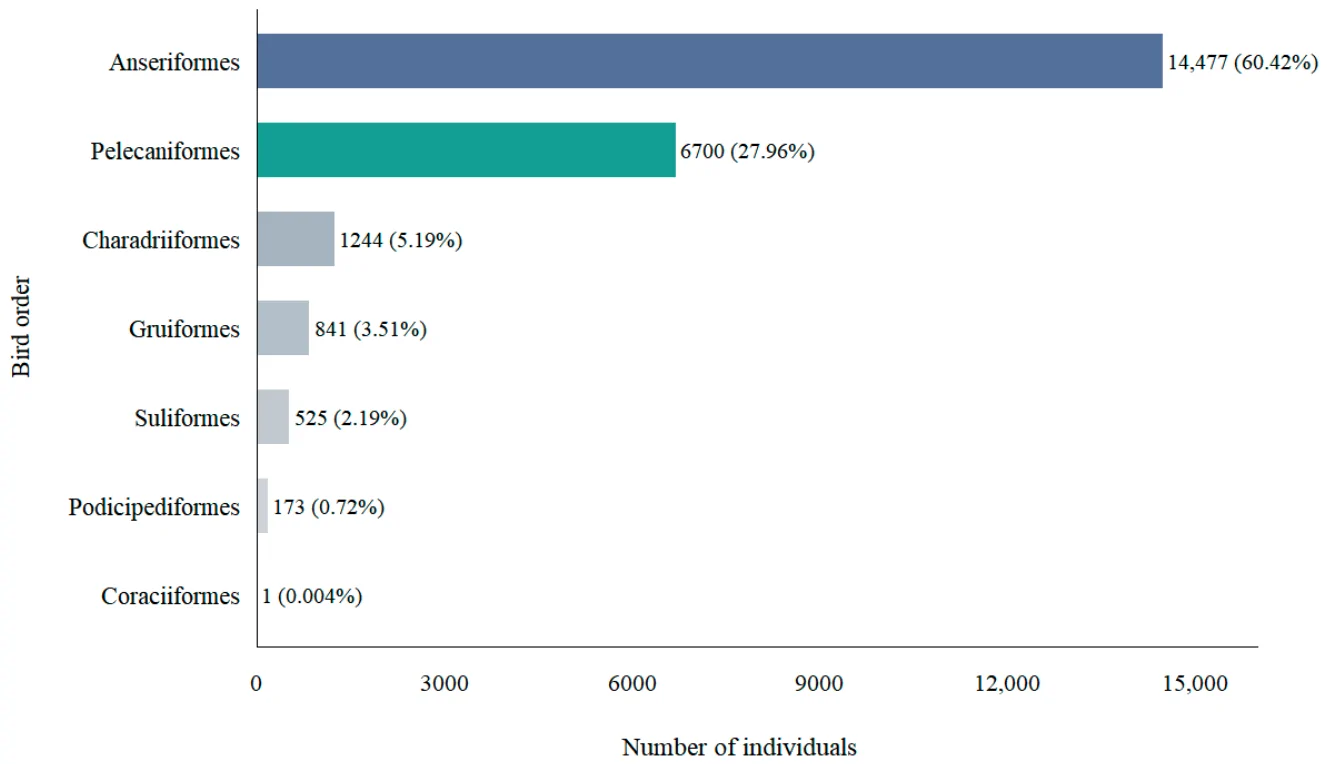
Composition and proportion of individuals in waterbird orders.

In total, 23,961 waterbirds were recorded (Supplementary Table S5 and Figure 3). Anseriformes had the largest population of 14,477 individuals, accounting for 60.42% of the total population, followed by Pelecaniformes, with 6700 individuals (27.96%). Charadriiformes and Gruiformes accounted for 5.19% and 3.51% of the total, respectively, while the remaining waterbird species accounted for 2.914%.

**Figure 4:**
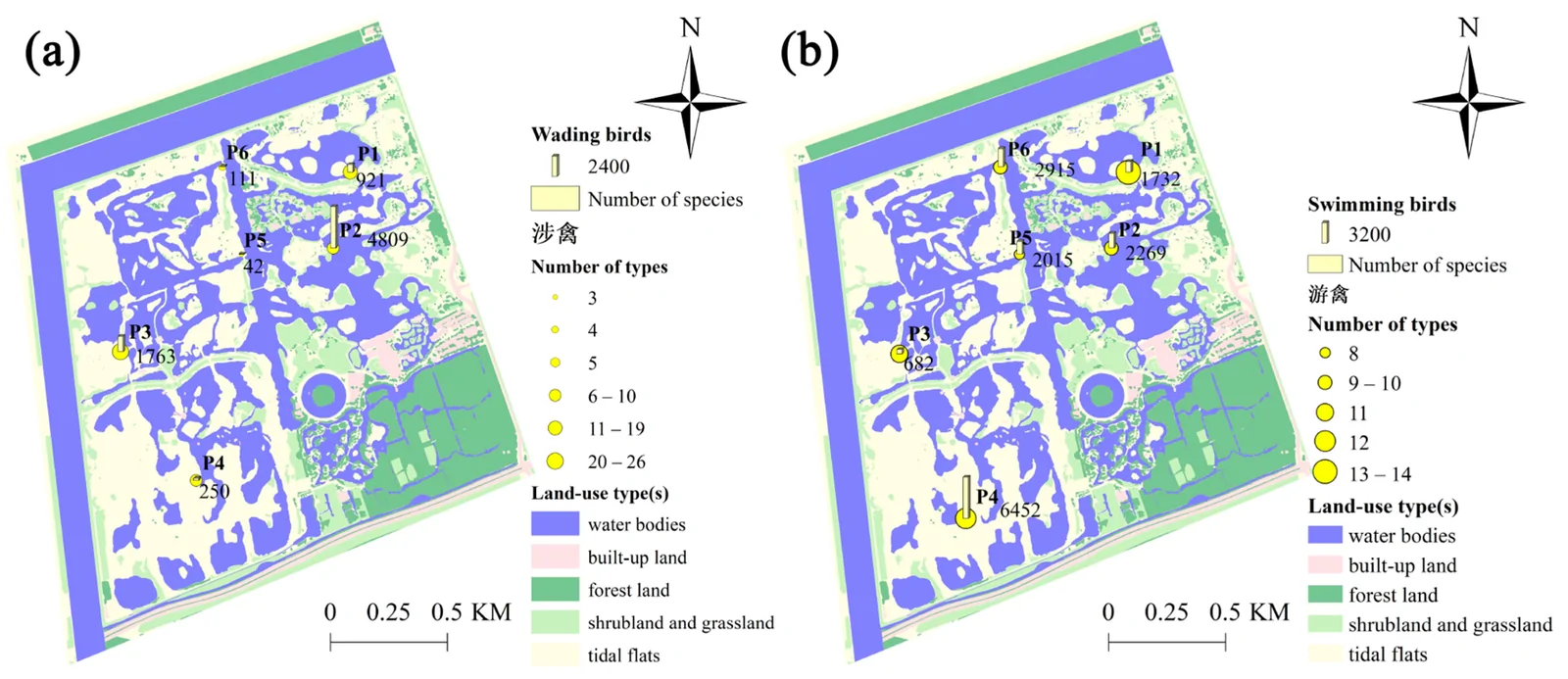
Spatial distribution of the six waterbird survey sites (P1–P6), species richness, and abundance across the study area: (**a**) wading birds and (**b**) swimming birds. The height of each bar indicates the number of individuals recorded at the corresponding survey site, whereas circle size indicates the number of species recorded. The base map shows five land-use types: forest land, shrub–grassland, tidal flat, built-up land, and water bodies. 涉禽: wading birds; 游禽: swimming birds.

Figure 4 illustrates the spatial distribution of species richness and number of wading and swimming birds in the study area. The number of wading bird species at each observation point ranged from 3 to 26, with the western part of the study area (P3) having a maximum of 26 species, followed by the northeastern part (P1), while the central river channel had the fewest species. The abundance of wading birds at each observation point ranged from 42 to 4809, showing a spatial pattern of “low in the center and high in the surrounding areas.” The eastern part (P2) had the largest population of wading birds, followed by the western part, and the fewest wading birds were recorded at P6. The number of swimming bird species at each observation point ranged from 8 to 14, with the northeastern part of the study area (P1) having the maximum of 14 species, followed by the southwestern part (P4), whereas the central river channel had fewer species. The abundance of swimming birds at each observation point ranged from 682 to 6452, presenting an overall spatial pattern in which the southwestern part (P4) had the largest population, followed by the northern and eastern parts, and the fewest swimming birds were recorded in P3.

### 3.3. Weight of Evaluation Factors

The analytic hierarchy results showed that the CR of the judgment matrix was 0.0054 (CR < 0.1), indicating a reasonable weight distribution. The weights of the evaluation factors are listed in Table 1. Among the first-level factors, food abundance had the highest weight, followed by water source condition, whereas the weights of disturbance condition and shelter degree were lower. Among the second-level factors, the water area had the highest weight for the three indicators under water source conditions, and the distance to the water source had the lowest weight. For the two indicators under disturbed conditions, the weight of the distance to visitor stopping points was greater than that of the distance to roads. Among the four indicators under shelter degree, patch number and vegetation coverage had the highest weights, whereas vegetation height and slope had the lowest weights. Regarding the food abundance factor, animal- and plant-derived food were the same weight, serving as the main food sources for wading and swimming birds, respectively [26,38,39].

### 3.4. Validation of Habitat Suitability Assessment

**Figure 5:**
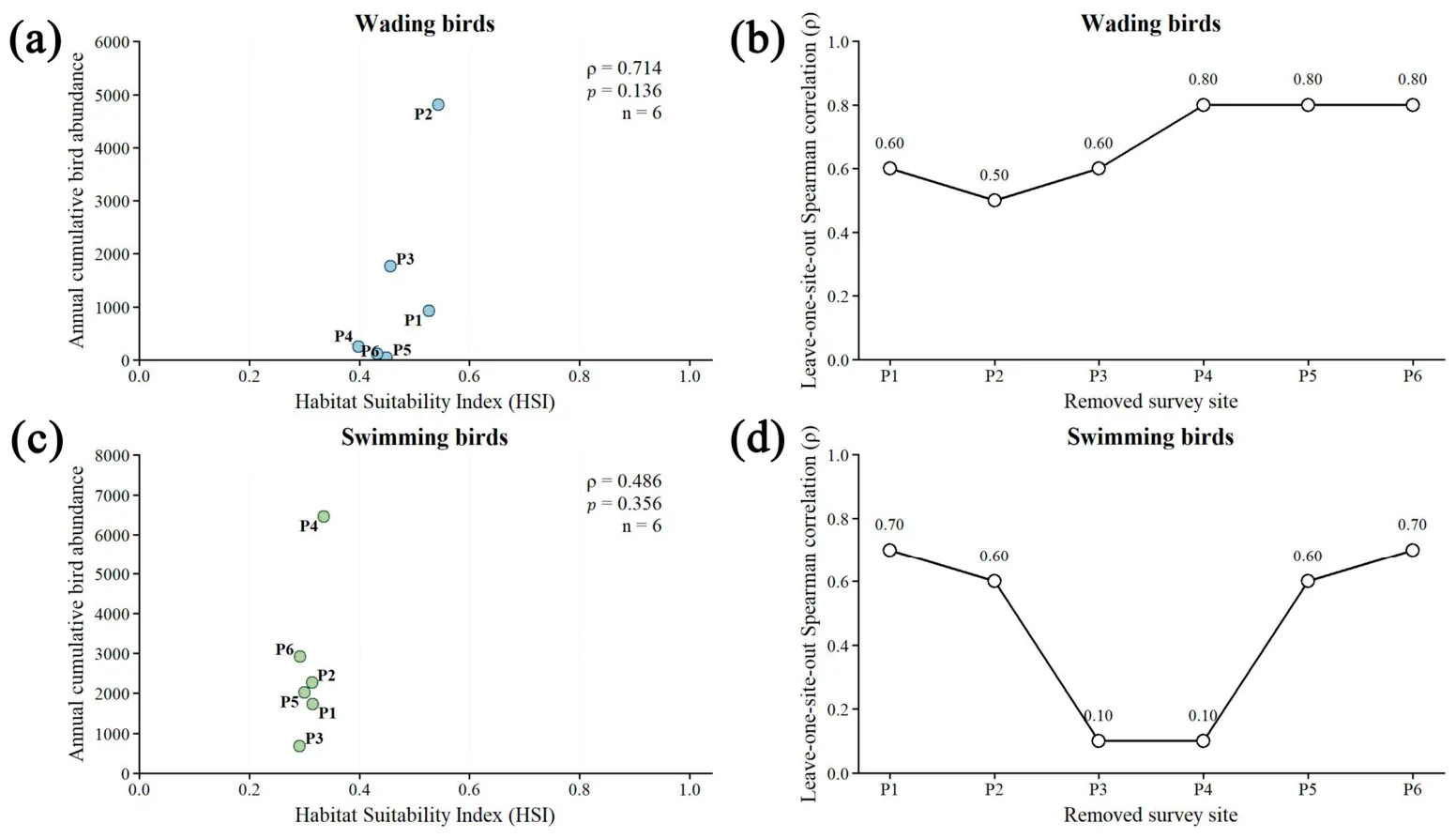
Annual-scale validation and leave-one-out sensitivity analysis of the habitat suitability index (HSI)–bird abundance relationships for wading and swimming birds. (**a**,**c**) Relationships between the HSI and annual cumulative bird abundance at the six survey sites for wading birds (**a**) and swimming birds (**c**). Each point represents one survey site; Spearman’s rank correlation coefficient (ρ) and the corresponding exact permutation *p* value are shown in each panel. (**b**,**d**) Leave-one-out (LOO) sensitivity analysis for wading birds (**b**) and swimming birds (**d**). Each point represents the Spearman correlation coefficient recalculated after excluding the indicated survey site and using the remaining five sites, with the corresponding LOO ρ value shown for each site.

At the annual scale, HSI was found to be positively correlated with cumulative bird abundance for both wading and swimming birds, with a stronger association for wading birds (Spearman’s ρ = 0.714, *p* = 0.136; Figure 5a) than for swimming birds (ρ = 0.486, *p* = 0.356; Figure 5c). The annual-scale LOO sensitivity analysis further showed that the correlation remained positive after removal of any single survey site, with ρ ranging from 0.50 to 0.80 for wading birds and from 0.10 to 0.70 for swimming birds (Figure 5b,d), indicating that both wading and swimming birds showed positive associations with HSI across different site combinations. As shown in Table 2, most permutation *p*-values across all months for both waterbird groups exceeded 0.05, thus indicating that the statistical significance criterion was not met. Nevertheless, the consistently positive effect sizes across the annual, monthly, and sensitivity analyses provide preliminary evidence that the HSI reflects spatial variation in waterbird habitat use to some extent, with comparatively better and more stable performance for wading birds.

### 3.5. Spatial Distribution Characteristics of Waterbird Habitat Suitability

**Figure 6:**
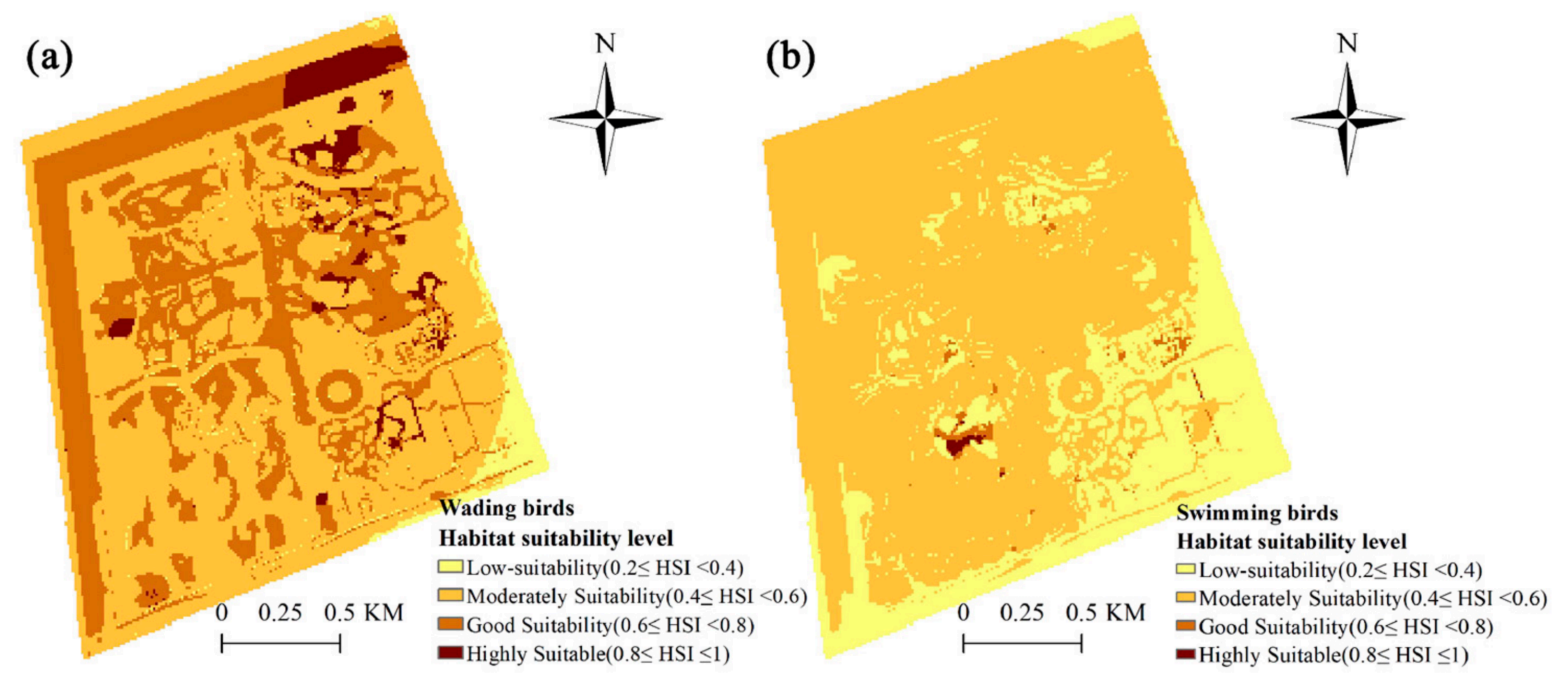
Spatial distribution of habitat suitability in the study area for (**a**) wading birds and (**b**) swimming birds. The mapped habitats comprise Grades II–V; no Grade I (unsuitable) habitat was identified for either group.

The spatial distribution maps of habitat suitability for wading and swimming birds in the study area are shown in Figure 6. The spatial distribution of habitat suitability differed between the wading and swimming birds. No unsuitable habitats were found for either group of birds [1,40,41], indicating that the overall environmental conditions were favorable and provided a strong habitat base. For the habitat suitability assessment of wading birds, the Grade III habitat had the largest area of 242.49 ha, accounting for 61.07% of the total area, and was mainly distributed in the land areas. This was followed by a Grade IV habitat, which was mainly located in the water areas. Grade V habitats were mainly distributed in the eastern part of the study area. Grade II habitats had the smallest area and were mainly distributed in woodland and construction land areas in the eastern part of the study area.

For the habitat suitability assessment of swimming birds, Grade III habitats in the study area had the largest area (290.97 ha), accounting for 73.27% of the total study area, and were mainly distributed in non-forest areas. Grade II habitats covered an area of 102.42 ha, accounting for 25.79%, and were mainly distributed in the southeastern woodland and areas close to roads. The areas of Grade IV and Grade V habitats were 3.02 ha and 0.71 ha, accounting for 0.76% and 0.18%, respectively, and they were mainly located in the pond areas in the southwestern part of the study area. In general, the study area was dominated by Grade II and Grade III habitats, which together accounted for 99.06% of the total area. This indicated that the overall habitat quality of the study area was at a moderate suitability level, with few habitats at extreme suitability grades.

**Figure 7:**
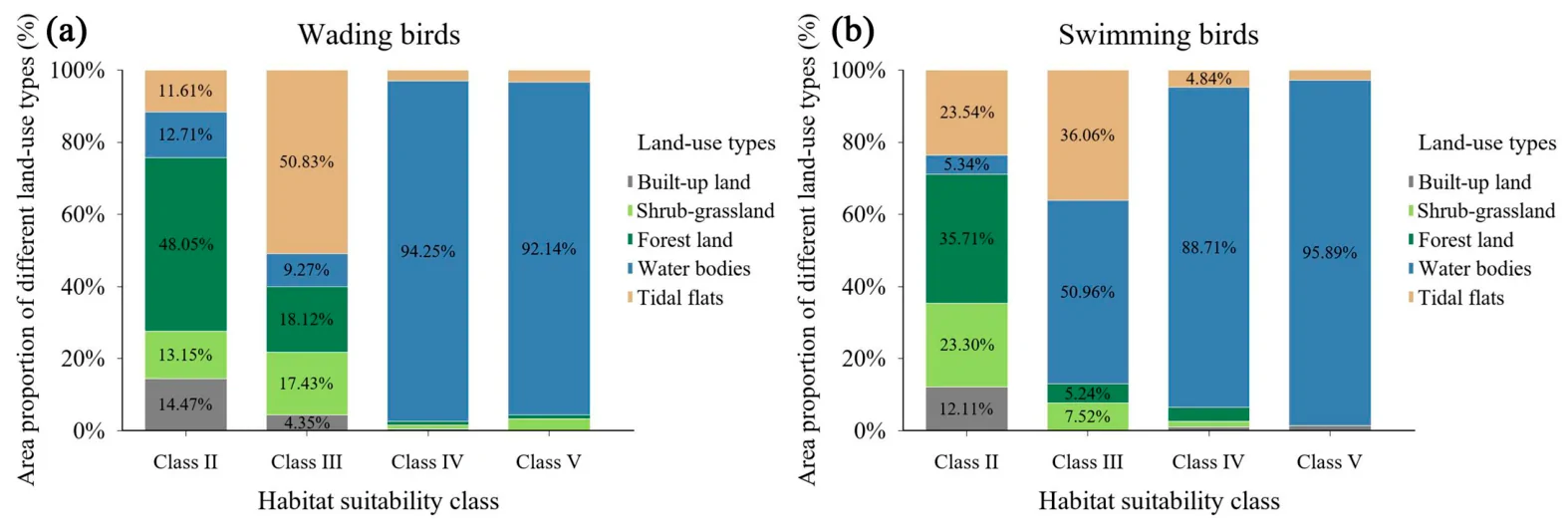
Composition of land-use types within different habitat-suitability grades for (**a**) wading birds and (**b**) swimming birds. Each bar shows the area occupied by the different land-use types within the corresponding habitat-suitability grade.

By overlaying the habitat suitability grade maps of wading and swimming birds with the spatial distribution map of land use, the land use conditions for each grade of habitat were obtained (Figure 7). Both Grade IV and V habitats of wading birds exhibited evident water-dominated features. Areas with Grade III habitat suitability were dominated by tidal flats, followed by woodlands, whereas water areas, shrub–grasslands, and other land types also accounted for a certain proportion, forming a wetland habitat with a composite structure [42]. Water areas accounted for an extremely high proportion of Grade IV and V habitats of swimming birds. Grade III habitats were dominated by water areas, accompanied by a relatively high proportion of tidal flats, forming a relatively complete habitat structure. In Grade II habitats, woodlands accounted for the highest proportion, followed by shrub–grasslands and tidal flats, whereas water areas had the lowest proportion. This indicates that low-grade habitats for swimming birds were mostly distributed in areas with relatively high degrees of terrestrial vegetation coverage, tidal flats, and human utilization.

## 4. Discussion

### 4.1. Differentiated Habitat Suitability Assessment for Wading and Swimming Birds

To construct the HSI model, we adopted a parameterization scheme characterized by consistent weights but differentiated grading criteria. Specifically, the wading and swimming birds were assigned the same factor weights, whereas separate grading criteria were established for the two functional groups (Supplementary Tables S1 and S2). The factor weights and grading criteria serve different functions in the evaluation model. The weights represent the relative contribution of each factor to waterbird habitat suitability and were assumed to remain relatively stable under the same environmental context within the study area for the two functional groups. In contrast, the grading criteria reflect differences among waterbird groups in terms of suitability thresholds for the same environmental factors [15,25,43]. Wading and swimming birds differ markedly in terms of their vegetation structure requirements, foraging strategies, and other ecological characteristics. Likewise, studies on wintering waterbirds in Poyang Lake have shown that different waterbird functional groups exhibit different response thresholds to mudflat, water, and vegetation areas [44]. Therefore, the use of common indicators and weights provided a consistent evaluation baseline. Differentiated suitability criteria retained differences in the ecological requirements of the two groups, thereby distinguishing between variations in the relative importance of habitat factors and variations in the ecological suitability ranges of the different waterbird types. Compared with conventional HSI approaches developed for individual species or target groups, this framework provides an additional means of facilitating cross-group comparisons under a common evaluation baseline. Accordingly, the “consistent weights and differentiated grading criteria” design in this study was primarily used to provide a common evaluation baseline for the comparisons between different waterbird functional groups, rather than being based on the assumption that the two groups respond identically to habitat factors. The ecological differences in habitat requirements between the two functional groups were mainly represented by group-specific grading criteria and suitability thresholds. By maintaining the comparability of the assessment results while retaining the ecological differentiation between functional groups, this framework provides a complementary approach for extending habitat suitability assessments from a single target group to differentiate comparisons among different ecological functional groups.

This differentiated parameterization is also reflected in specific habitat factors. In the present study, the grading criteria for food types differed substantially between wading and swimming birds. Animal-derived food is the main food source for wading birds, and shorebirds with different bill lengths achieve niche differentiation by feeding on benthic animals at different depths and particle sizes [44]. The diets of swimming birds are dominated by plant-derived foods, among which Anatidae species consume hygrophytic vegetation such as sedges as their main food source. Although swimming birds feed opportunistically on benthic animals and fish, plant buds and seeds remain dominant in their diets [45,46]. The water depth standards for highly suitable habitats for swimming and wading birds also differ. Wading birds have long legs and slender toes and rely on vision to search for prey in shallow water or bare tidal flats. Swimming birds have short legs and webbed toes and rely on water buoyancy to swim or dive for foraging. Increasing water depth significantly reduces the abundance of wading birds but increases that of swimming birds [47,48]. In terms of vegetation structure, this study set the vegetation coverage of the highly suitable habitat for wading birds at 0–35% and that for swimming birds at 45–70%. This difference stems from the different functional demands of the two waterbird groups on vegetation structure. Wading birds need to forage in open shallow water or bare tidal flats, and excessively tall and dense vegetation blocks their field of vision, reduces foraging efficiency, and reduces their ability to detect predators. By contrast, swimming birds require vegetation with moderate coverage to provide shelter between the water surface and land without hindering their activities. In terms of human disturbance factors, based on the actual situation in the study area, highly suitable distances for wading birds to avoid road and tourist disturbances were set as >220 m and >450 m, respectively, whereas those for swimming birds were set as >250 m and >520 m, respectively. The tolerance distance of wading birds was significantly smaller than that of swimming birds, which may be related to the flight initiation distance and behavioral adaptation strategies of the different waterbird groups [49].

### 4.2. Relative Importance of Environmental Factors for Waterbird Habitat Suitability

The analytic hierarchy process results showed that the weights of each evaluation factor differed significantly (see Supplementary File “Questionnaire” for details). Among the first-level factors, food abundance had the highest weight, followed by water source and disturbance conditions, while shelter degree had relatively low importance. This ranking reflected the degree of restriction on each environmental factor in the study area for waterbird habitat selection. The highest weight of food abundance indicates that the availability of food resources is the primary factor affecting the distribution of waterbirds in the study area. This result is broadly consistent with previous results from coastal wetlands in the Bohai Rim, which have shown that variables closely associated with food webs, such as vegetation cover and macroinvertebrate diversity, can significantly explain variations in waterbird diversity among different wetlands [14,50,51]. Located in the middle section of the East Asian–Australasian Flyway, Hangzhou Bay wetland is a critical stopover site for migratory waterbirds. Migratory waterbirds use the site primarily to replenish their energy reserves; therefore, the spatiotemporal distribution of food resources directly determines the residence time and habitat utilization intensity of waterbirds.

The weight of water source condition ranked second only to food abundance because waterbird behaviors, such as foraging, drinking, and preening, depend on the presence of water bodies [52]. Among the indicators of water source conditions, the weight of water area was higher than that of water depth and distance to the water source. A larger water area not only provides broader spaces for foraging, roosting, and disturbance avoidance but also helps maintain stable hydrological processes and a high carrying capacity of food resources. Although the weights of water depth and distance to water sources are relatively low, water depth is also a key factor affecting the habitat preferences of wading and swimming birds [53,54]. During the migratory stopover period, areas close to water sources can reduce energy consumption for waterbirds, thereby supporting higher frequencies of activity. This is also the case in floodplain wetlands with pronounced seasonal water level fluctuations, such as Poyang Lake. Changes in water level continuously alter the proportions of deep-water areas, shallow-water zones, mudflats, and vegetated habitats, resulting in relatively distinct hydrological threshold responses among different waterbird functional groups [55]. However, as the water bodies in the study area are mainly artificially regulated ponds and ditches with a relatively uniform distribution, the limiting effect of water source conditions on habitat suitability is slightly lower than that of food resources.

The weights of the disturbance conditions and shelter degree are relatively low because the wetland park has implemented relatively effective control measures (such as restricting tourist activity areas and setting up buffer zones) to limit the intensity of human disturbance of waterbirds [56], and the study area has good vegetation coverage that provides shelter conditions for waterbirds. Among the disturbance condition indicators, the weight of distance to visitor stopping points was greater than that of distance to roads. This is mainly because roads represent stable disturbances to which birds may habituate [57], whereas tourist disturbances are spatially concentrated and unpredictable, making them more likely to trigger vigilance and avoidance behaviors in waterbirds [58,59]. For shelter degree indicators, vegetation coverage and patch number had greater effects, as these two factors directly determine the behavioral availability of habitats for waterbirds. For wading birds, low vegetation coverage and a reasonable patch pattern can ensure an open field of vision and favorable accessibility, thus balancing foraging efficiency and safety. For swimming birds, vegetation coverage and patch connectivity determine the quality of shelter conditions and affect perceived safety at roosting sites [60]. In the present study, the weights of slope and vegetation height were relatively low, probably because the study area was located in the core zone of the wetland park, which has gentle terrain. The vegetation height classification was based on the actual height of the waterbird distribution and existing vegetation conditions. The vegetation of all grades was spatially distributed, with no significant overall differences.

The identified factors, including food availability, water resources, disturbance, and shelter conditions, are among the most recognized habitat drivers in studies of East Asian wetland waterbirds. However, their relative importance varies among regions. Studies conducted in wetlands such as Yancheng, Bohai Rim, and Poyang Lake have emphasized the roles of human disturbance, benthic food resources, hydrological variation, and vegetation structure [51,55,61], whereas food resources and water availability were more prominent in the present study. This difference indicates that the dominant factors determining waterbird habitat suitability are strongly site dependent. Their effects are not only determined by the ecological requirements of the waterbirds themselves but are also jointly shaped by regional contexts, including wetland type, hydrological processes, conservation management intensity, and levels of human activity.

### 4.3. Spatial Patterns of Waterbird Habitat Suitability and Ecological Interpretation

The results showed that there were significant differences in the spatial distribution of habitat suitability between wading and swimming birds in the Hangzhou Bay National Wetland Park, which reflected the differences in wetland resource-use patterns and habitat demands between the waterbird groups. This spatial differentiation is mainly affected by the combined effects of environmental factors such as water source conditions, land-use type, vegetation structure, and food resources. Different waterbird communities achieve niche differentiation by utilizing different habitat resources [53] to reduce interspecific competition and improve the stability and diversity of the wetland waterbird community. This result is consistent with the findings of domestic studies on waterbird habitat suitability in coastal wetlands, indicating that the spatial pattern of waterbird habitat suitability is comprehensively affected by multiple environmental factors, such as water condition, food resources, and human disturbance [28]. Similar temporal responses have been reported for other wetland systems. Studies conducted on Korean tidal flats and Poyang Lake have shown that the spatial distribution of waterbirds can shift substantially in response to seasonal changes in water levels, food resources, and vegetation conditions [55,62]. Given the limited temporal resolution of several environmental variables, the present analysis has focused on the overall spatial pattern of habitat suitability rather than on seasonal variation.

From the perspective of spatial patterns, habitats with moderate and high suitability accounted for a large proportion of the study area, while highly suitable areas were mainly distributed in continuous water bodies, tidal flats, and well-preserved wetlands. Although the two groups were evaluated using the same land-use dataset, there were significant differences in the distribution of the same land-use type across HSI grades (Figure 7). This therefore indicates that the habitat suitability of the two waterbird groups does not simply depend on land-use type but is formed by the combined effect of different ecological demands and environmental factors. The habitats of wading birds in the water areas were mainly classified as Grades IV and V, whereas those of swimming birds in the water areas were mostly classified as Grade III. This difference was mainly related to the distinct spatial distribution of food resources between waterbird groups. Wading birds are highly dependent on animal-derived food resources, and their suitable habitats usually correspond to shallow-water areas, mudflats, and aquatic–terrestrial ecotones rich in benthic invertebrates and small aquatic animals [11,40]. Therefore, the water areas with a high density of animal-derived food and favorable foraging conditions are more likely to be classified as highly suitable habitats for wading birds. In contrast, habitat suitability for swimming birds was affected by the distribution of aquatic plants, plant seeds, and other plant-derived food resources. Although some water areas in our study can provide basic stopover points for swimming birds, the coverage of aquatic plants and the abundance and accessibility of plant-derived food resources may not reach a high level; therefore, most water areas were classified as Grade III. It is clear that the water area itself cannot fully determine the habitat suitability of waterbirds. Therefore, the type, availability, and accessibility of food resources within water bodies may explain the differences in habitat suitability grades.

In recent years, Hangzhou Bay National Wetland Park has continuously promoted wetland conservation and ecological restoration. Through measures such as tidal creek restoration, vegetation rehabilitation, and natural shoreline protection, the integrity of wetland ecosystems has been improved. Public monitoring data show that by 2025, 303 bird species will be recorded in the wetland park, including multiple species under national protection. The number of migratory and wintering waterbirds remained high every year, thus indicating that the study area has a favorable wetland ecological carrying capacity. This is consistent with the results of this study, in which habitats with moderate and high suitability accounted for a large proportion.

### 4.4. Limitations and Future Directions

The present study was designed to characterize the overall spatial patterns of habitat suitability for wading and swimming birds during the study period, rather than the short-term seasonal dynamics. Accordingly, the HSI values obtained in this study should be interpreted as relative measures of overall habitat suitability across different areas during the study period, rather than as a complete characterization of habitat suitability for specific months or seasons. This interpretation is also constrained by the temporal resolution of the environmental data: although waterbird surveys were conducted monthly, several environmental variables were unavailable at comparable temporal resolutions, limiting the capacity of the HSI model to represent month-to-month or seasonal variation. This constraint is particularly relevant to food resources, which vary substantially across space and time; the food-resource indicators used here are more appropriately interpreted as measures of relative spatial availability rather than as representations of short-term dynamics or absolute resource stocks. The model evaluation was also limited by the small number of independent survey sites. Spearman’s correlation, monthly consistency, and leave-one-site-out sensitivity analyses indicated spatial agreement between predicted habitat suitability and observed waterbird use; however, the available evidence remains insufficient for statistically robust validation of predictive performance. Under these aforementioned conditions, the model is more appropriately regarded as providing indicative evidence of habitat suitability patterns within the study area, with particular caution required when interpreting the results for swimming birds because of the limited independent validation data. More seasonally resolved environmental data, a larger set of independent survey sites, and multiyear observations would provide a stronger basis for evaluating temporal variability, predictive robustness, and model generalizability.

### 4.5. Recommendations for Conservation and Management of Hangzhou Bay Wetlands

As a stopover site for waterbirds and a key biodiversity area, Hangzhou Bay provides a valuable basis for proposing recommendations for wetland conservation and management. Food availability was identified as the primary factor limiting waterbird distribution. Therefore, in the core distribution area of swimming birds (P4), maintaining relatively high water levels should be prioritized to ensure suitable deep-water foraging conditions. In the core distribution area of wading birds (P2), particular attention should be given to maintaining a stable supply of animal-based food resources, such as fish and shrimp. Human disturbance is most pronounced in P4, which also supports the highest concentration of swimming birds. Therefore, vegetation buffer zones and ecological isolation belts should be established in combination with appropriate spatial management measures to reduce the persistent impact of human activities on swimming bird habitats. In contrast, areas preferred by wading birds currently experience lower levels of disturbance, a condition that should be maintained by avoiding the introduction of new roads, recreational facilities, or other high-intensity human activities. Regarding vegetation management, in swimming bird core areas, certain reed patches and other vegetation structures should be retained while preventing excessive expansion of tall emergent plants, as continuous vegetation encroachment may reduce open-water areas and available foraging habitats. In wading bird core areas, the spatial continuity and structural stability of tall vegetation habitats, such as *Arundo donax* communities, should be maintained, and human disturbance should be minimized. Appropriate vegetation coverage and height should be maintained to provide suitable conditions for wading bird nesting, thereby improving nest concealment and breeding safety. To this end, bird monitoring and habitat management should be strengthened, and differentiated management strategies should be implemented according to habitat suitability assessments. In highly suitable areas, the focus should be on strict protection and maintenance of existing high-quality habitats. Moderately suitable areas should undergo ecological optimization targeting their major limiting factors. Low-suitability areas should be subject to targeted habitat restoration based on the key environmental factors restricting their suitability [63,64]. However, these recommended measures should be validated through management experiments or long-term monitoring to verify their effectiveness for future conservation practices. Meanwhile, the publicity and education of ecological conservation must be strengthened for tourists and residents through standardized activities such as bird watching and photography, which will enhance the public’s awareness of and participation in wetland conservation.

## 5. Conclusions

This study was conducted in the educational and exhibition areas of the Hangzhou Bay National Wetland Park. A differentiated habitat suitability model was developed for wading birds and swimming birds, based on bird survey data from 2025 and remote sensing imagery, and the model was evaluated. Analysis of the spatial distribution characteristics of suitable habitats for wading and swimming birds showed that food resources and water availability were the dominant factors affecting waterbird habitat suitability, whereas concealment conditions and disturbance levels had relatively weaker effects. The HSI values were positively correlated with the number of observed waterbirds, indicating that the HSI-based model reflected the actual distribution patterns of waterbirds to some extent. Overall, waterbird habitats exhibited relatively high suitability, with most areas classified as moderately suitable or above. No extremely unsuitable areas were identified. The differentiated HSI evaluation framework provided a methodological reference for assessing waterbird habitat suitability in small-scale wetland protected areas. Future studies should incorporate hydrological, vegetation, and food resource data from different periods, such as migration and winter seasons, and integrate long-term monitoring datasets and comparisons among multiple models to further evaluate the stability and applicability of the model.

## Figures and Tables

**Table 1:** Factor weights of waterbird habitat suitability assessment.

Criterion-Layer Factor	Weight	Indicator-Layer Factor	Weight
Water condition	0.2718	Water depth	0.0848
		Distance to water source	0.0537
		Water area	0.1333
Disturbance condition	0.1575	Distance to road	0.0394
		Distance to visitor stopping point	0.1181
Concealment	0.0883	Vegetation cover	0.0239
		Number of patches	0.0373
		Vegetation height	0.0143
		Slope	0.0127
Food abundance	0.4824	Animal-derived food/plant-derived food	0.4824

**Table 2:** Spearman correlations between the HSI value and monthly bird abundance for wading and swimming birds.

Month	Wading Birds		Swimming Birds	
	Spearman’s ρ	*p* Value	Spearman’s ρ	*p* Value
January	0.683	0.2	0.371	0.497
February	−0.353	0.522	0.371	0.497
March	0.638	0.2	0.543	0.297
April	0.829	0.058	−0.257	0.658
May	0.886	0.033	−0.058	0.939
June	0.754	0.106	0.273	0.617
July	0.812	0.072	0.759	0.117
August	0.543	0.297	0.754	0.111
September	0.667	0.161	0.714	0.136
October	0.638	0.200	0.348	0.494
November	0.486	0.356	0.314	0.564
December	0.486	0.356	0.429	0.419

## Data Availability

The datasets used and analyzed during the current study are available from the corresponding author.

## Supplementary Materials

The following supporting information can be downloaded at: https://www.mdpi.com/article/10.3390/biology15181617/s1. Table S1: Grading criteria for habitat suitability factors for wading birds; Table S2: Grading criteria for habitat suitability factors for swimming birds; Table S3: Meaning of the Saaty scale; Table S4: Classification criteria for the habitat suitability index; Table S5: List of bird species; Questionnaire: Survey on Factor Weights for Waterbird Habitat Suitability. References [65,66,67,68] are cited in the Supplementary Materials.
