# Supplementary material for: A Detailed Evaluation of the Suitability of Waterbird Habitats in Hangzhou Bay National Wetland Park—A Differential Analysis of Wading Birds and Swimming Birds
Source: Biology (Basel). 2026 Sep 14;15(18):1617. doi: 10.3390/biology15181617 (PMC13603968; doi:10.3390/biology15181617)
Supplement: Supplementary file 1 [file biology-15-01617-s001.zip › Supplementary Materials.pdf]

Table S1. Grading criteria for habitat suitability factors for wading birds

Code	Criterion layer	Indicator layer	Highly suitable (1)	Good suitability (0.8)	Moderately suitable (0.6)	Low suitability (0.4)	Unsuitable (0.2)
1	Water condition	Water depth (cm) [18,52,53]	0–5	5–10	10–20	20–30	>30
2		Water area (ha) [25,43,50]	>10	5–10	2–5	0.5–2	0–0.5
3		Distance to water source (m) [15,25]	0–30	30–60	60–100	100–150	>150
4	Concealment	Vegetation cover (%) [15,50]	0–35	35–40	40–50	50–65	>65
5		Vegetation height (m) [19]	1.45–1.6	1–1.45	0–1	1.6–1.7	>1.7
6		Number of patches [42]	70–120	120–150	30–70	150–170	0–30
7		Slope (°) [28]	<1	1–3	3–5	5–10	>10
8	Disturbance condition	Distance to road (m) [15,59]	>220	150–220	80–150	30–80	0–30
9		Distance to visitor stopping point (m) [49]	>450	280–450	150–280	60–150	0–60
10	Food abundance	Animal-derived food (g·trap^{-1}) [20,21,26,50,65]	>800	600–800	400–600	200–400	<200

Table S2. Grading criteria for habitat suitability factors for swimming birds

Code	Criterion layer	Indicator layer	Highly suitable (1)	Good suitability (0.8)	Moderately suitable (0.6)	Low suitability (0.4)	Unsuitable (0.2)
1	Water condition	Water depth (cm) [66]	>60	40–60	20–40	10–20	<10
2		Water area (ha) [9]	>15	8–15	3–8	1–3	<1
3		Distance to water source (m) [15]	0–50	50–100	100–140	140–180	>180
4	Concealment	Vegetation cover (%) [9,15,24]	45–70	30–45	70–80	15–30	<15 or >80
5		Vegetation height (m) [67,68]	1.3–1.7	1.7–2	0.8–1.3	2–3	<0.8 or >3
6		Number of patches [42]	70–160	160–260	260–350	0–70	>350
7		Slope (°) [9]	<1	1–3	3–5	5–10	>10
8	Disturbance condition	Distance to road (m) [9,15,57]	>250	180–250	120–180	50–120	0–50
9		Distance to visitor stopping point (m) [49,56,58]	>520	350–520	200–350	80–200	0–80
10	Food abundance	Plant-derived food [9,38,45,46]	0.8–1	0.6–0.8	0.4–0.6	0.2–0.4	0–0.2

Table S3. Meaning of the Saaty scale

Scale	Meaning	Explanation
1	Equal importance	The two factors exert equal influence on the target.
3	Slightly more important	The former factor is slightly more important than the latter with respect to the target.
5	Obviously more important	The former factor is significantly more important than the latter with respect to the target.
7	Strongly more important	The former factor is much more important than the latter with respect to the target.
9	Extremely more important	The former factor is overwhelmingly more important than the latter with respect to the target.
2, 4, 6, 8	Intermediate values	Used as intermediate values between adjacent judgments.
Reciprocals (1/3, 1/5, …, 1/9)	Reverse comparison	Indicates the importance of the latter factor relative to the former with respect to the target.

Table S4. Classification criteria for the habitat suitability index

Grade	HSI Range	Habitat suitability grade	Ecological significance
I	0 ≤ HSI < 0.2	Unsuitable	Poor habitat conditions that hardly meet the basic habitat requirements of waterbirds.
II	0.2 ≤ HSI < 0.4	Low suitability	Only partially meets some habitat requirements, with prominent limiting factors.
III	0.4 ≤ HSI < 0.6	Moderately suitable	Meets the general habitat requirements of waterbirds but remains subject to certain constraints.
IV	0.6 ≤ HSI < 0.8	Good suitability	Favorable habitat conditions that meet the main survival and activity requirements of waterbirds.
V	0.8 ≤ HSI ≤ 1	Highly suitable	Superior habitat conditions that fully meet the roosting, foraging, and breeding requirements of waterbirds.

Table S5. List of bird species

Code	Order	Family	Common name	Scientific name	National protection class (China)	IUCN category
1	Podicipediformes	Podicipedidae	Little Grebe	*Tachybaptus ruficollis*	—	LC
2	Podicipediformes	Podicipedidae	Great Crested Grebe	*Podiceps cristatus*	—	LC
3	Suliformes	Phalacrocoracidae	Great Cormorant	*Phalacrocorax carbo*	—	LC
4	Pelecaniformes	Ardeidae	Grey Heron	*Ardea cinerea*	—	LC
5	Pelecaniformes	Ardeidae	Purple Heron	*Ardea purpurea*	—	LC
6	Pelecaniformes	Ardeidae	Great Egret	*Ardea alba*	—	LC
7	Pelecaniformes	Ardeidae	Medium Egret	*Ardea intermedia*	—	LC
8	Pelecaniformes	Ardeidae	Little Egret	*Egretta garzetta*	—	LC

9	Pelecaniformes	Ardeidae	Cattle Egret	*Bubulcus ibis*	—	LC
10	Pelecaniformes	Ardeidae	Chinese Pond Heron	*Ardeola bacchus*	—	LC
11	Pelecaniformes	Ardeidae	Black-crowned Night Heron	*Nycticorax nycticorax*	—	LC
12	Pelecaniformes	Ardeidae	Yellow Bittern	*Ixobrychus sinensis*	—	LC
13	Pelecaniformes	Threskiornithidae	Eurasian Spoonbill	*Platalea leucorodia*	Class II	VU
14	Pelecaniformes	Threskiornithidae	Black-faced Spoonbill	*Platalea minor*	Class I	EN
15	Anseriformes	Anatidae	Falcated Duck	*Mareca falcata*	—	NT
16	Anseriformes	Anatidae	Garganey	*Spatula querquedula*	—	LC
17	Anseriformes	Anatidae	Eurasian Wigeon	*Mareca penelope*	—	LC
18	Anseriformes	Anatidae	Gadwall	*Mareca strepera*	—	LC
19	Anseriformes	Anatidae	Eurasian Teal	*Anas crecca*	—	LC
20	Anseriformes	Anatidae	Mallard	*Anas platyrhynchos*	—	LC
21	Anseriformes	Anatidae	Chinese Spot-billed Duck	*Anas zonorhyncha*	—	LC
22	Anseriformes	Anatidae	Northern Shoveler	*Spatula clypeata*	—	LC
23	Anseriformes	Anatidae	Tufted Duck	*Aythya fuligula*	—	LC
24	Anseriformes	Anatidae	Greylag Goose	*Anser anser*	Class II	LC
25	Anseriformes	Anatidae	Mandarin Duck	*Aix galericulata*	Class II	LC
26	Gruiformes	Rallidae	Common Moorhen	*Gallinula chloropus*	—	LC
27	Gruiformes	Rallidae	Eurasian Coot	*Fulica atra*	—	LC
28	Gruiformes	Rallidae	Ruddy-breasted Crake	*Porzana fusca*	—	LC
29	Gruiformes	Rallidae	White-breasted Waterhen	*Amaurornis phoenicurus*	—	LC
30	Charadriiformes	Charadriidae	Kentish Plover	*Charadrius alexandrinus*	—	LC
31	Charadriiformes	Charadriidae	Little Ringed Plover	*Charadrius dubius*	—	LC
32	Charadriiformes	Charadriidae	Grey-headed Lapwing	*Vanellus cinereus*	—	LC
33	Charadriiformes	Glareolidae	Oriental Pratincole	*Glareola maldivarum*	—	LC
34	Charadriiformes	Scolopacidae	Common Snipe	*Gallinago gallinago*	—	LC
35	Charadriiformes	Scolopacidae	Wood Sandpiper	*Tringa glareola*	—	LC
36	Charadriiformes	Scolopacidae	Common Sandpiper	*Actitis hypoleucos*	—	LC
37	Charadriiformes	Scolopacidae	Common Greenshank	*Tringa nebularia*	—	LC
38	Charadriiformes	Scolopacidae	Black-tailed Godwit	*Limosa limosa*	—	NT
39	Charadriiformes	Scolopacidae	Dunlin	*Calidris alpina*	—	LC
40	Charadriiformes	Scolopacidae	Temminck's Stint	*Calidris temminckii*	—	LC

41	Charadriiformes	Scolopacidae	Little Stint	*Calidris minuta*	—	LC
42	Charadriiformes	Scolopacidae	Sharp-tailed Sandpiper	*Calidris acuminata*	—	LC
43	Charadriiformes	Scolopacidae	Sanderling	*Calidris alba*	—	LC
44	Charadriiformes	Recurvirostridae	Pied Avocet	*Recurvirostra avosetta*	—	LC
45	Charadriiformes	Recurvirostridae	Black-winged Stilt	*Himantopus himantopus*	—	LC
46	Charadriiformes	Jacanidae	Pheasant-tailed Jacana	*Hydrophasianus chirurgus*	Class II	LC
47	Charadriiformes	Laridae	Little Tern	*Sternula albifrons*	—	LC
48	Charadriiformes	Laridae	Gull-billed Tern	*Gelochelidon nilotica*	—	LC
49	Charadriiformes	Laridae	Whiskered Tern	*Chlidonias hybrida*	—	LC
50	Coraciiformes	Alcedinidae	Common Kingfisher	*Alcedo atthis*	—	LC
