# Supplementary material for: A Detailed Evaluation of the Suitability of Waterbird Habitats in Hangzhou Bay National Wetland Park—A Differential Analysis of Wading Birds and Swimming Birds
Source: Biology (Basel). 2026 Sep 14;15(18):1617. doi: 10.3390/biology15181617 (PMC13603968; doi:10.3390/biology15181617)
Supplement: Supplementary file 1 [file biology-15-01617-s001.zip › Questionnaire.pdf]

Survey on Factor Weights for Waterbird Habitat Suitability

Question 1: Basic Information

Name:	
Organization / Affiliation:	
Specialty	

Questionnaire Instructions

This questionnaire uses the Analytic Hierarchy Process (AHP) to evaluate the relative importance of factors affecting waterbird habitat suitability. Based on your professional judgment, please select the corresponding level on the left or right for each pairwise comparison below:

1: Equally important

3: Slightly more important

5: Comparatively more important

7: Much more important

9: Absolutely more important

Instructions for completion:

1. Select only one score for each pairwise comparison and tick the corresponding box.
2. A score on the left indicates that the factor on the left is more important; a score on the right indicates that the factor on the right is more important.
3. If you cannot judge the relative importance of the two factors, select 1 (equally important).

Pairwise Comparison of Primary Factors

Description of factors:

Factor	Description
Water-source condition	The capacity of hydrological conditions in the study area to support waterbird habitat, including water-body distribution, water-area extent, and water-level conditions. It is an important fundamental factor affecting waterbird roosting and foraging.
Disturbance condition	The intensity of disturbance to waterbird habitat caused by human activities, mainly reflecting the effects of traffic activity, concentrations of residents, and other anthropogenic factors on waterbird behavior and habitat safety.
Shelter / cover	The capacity of terrain and land-use structure within the habitat to provide concealment, protection from disturbance, and improved habitat conditions for waterbirds.
Food abundance	The overall status of food resources available to waterbirds in the study area, including the spatial distribution and abundance of vegetation and benthic biological resources. It is one of the key factors determining waterbird habitat quality.

Question 2: Water-source condition VS Disturbance condition

Left absolutely more important (9)	Left much more important (7)	Left comparatively more important (5)	Left slightly more important (3)	Equally important (1)	Right slightly more important (3)	Right comparatively more important (5)	Right much more important (7)	Right absolutely more important (9)
☐	☐	☐	☐	☐	☐	☐	☐	☐

Question 3：Water-source condition VS Shelter / cover

Left absolutely more important (9)	Left much more important (7)	Left comparatively more important (5)	Left slightly more important (3)	Equally important (1)	Right slightly more important (3)	Right comparatively more important (5)	Right much more important (7)	Right absolutely more important (9)
☐	☐	☐	☐	☐	☐	☐	☐	☐

Question 4：Water-source condition VS Food abundance

Left absolutely more important (9)	Left much more important (7)	Left comparatively more important (5)	Left slightly more important (3)	Equally important (1)	Right slightly more important (3)	Right comparatively more important (5)	Right much more important (7)	Right absolutely more important (9)
☐	☐	☐	☐	☐	☐	☐	☐	☐

Question 5：Disturbance condition VS Shelter / cover

Left absolutely more important (9)	Left much more important (7)	Left comparatively more important (5)	Left slightly more important (3)	Equally important (1)	Right slightly more important (3)	Right comparatively more important (5)	Right much more important (7)	Right absolutely more important (9)
☐	☐	☐	☐	☐	☐	☐	☐	☐

Question 6：Disturbance condition VS Food abundance

Left absolutely more important (9)	Left much more important (7)	Left comparatively more important (5)	Left slightly more important (3)	Equally important (1)	Right slightly more important (3)	Right comparatively more important (5)	Right much more important (7)	Right absolutely more important (9)
☐	☐	☐	☐	☐	☐	☐	☐	☐

Question 7：Shelter / cover VS Food abundance

Left absolutely more important (9)	Left much more important (7)	Left comparatively more important (5)	Left slightly more important (3)	Equally important (1)	Right slightly more important (3)	Right comparatively more important (5)	Right much more important (7)	Right absolutely more important (9)
☐	☐	☐	☐	☐	☐	☐	☐	☐

Pairwise Comparison of Secondary Factors

Secondary Factors under "Water-source Condition"

Description of factors:

Factor	Description
Water depth	Reflects the depth or water-level conditions of water bodies in the study area. Different water depths affect the activity range and foraging methods of waterbirds and have different ecological significance for swimming and wading waterbirds.
Water-body area	The spatial extent of water bodies within the study area. A larger water surface generally provides more sufficient habitat and foraging space.
Distance to water source	The spatial distance from a habitat unit to the nearest water body. The shorter the distance, the easier it is for waterbirds to obtain water and food resources.

Question 8：Water depth VS Water-body area

Left absolutely more important (9)	Left much more important (7)	Left comparatively more important (5)	Left slightly more important (3)	Equally important (1)	Right slightly more important (3)	Right comparatively more important (5)	Right much more important (7)	Right absolutely more important (9)
☐	☐	☐	☐	☐	☐	☐	☐	☐

Question 9：Water depth VS Distance to water source

Left absolutely more important (9)	Left much more important (7)	Left comparatively more important (5)	Left slightly more important (3)	Equally important (1)	Right slightly more important (3)	Right comparatively more important (5)	Right much more important (7)	Right absolutely more important (9)
☐	☐	☐	☐	☐	☐	☐	☐	☐

Question 10：Water-body area VS Distance to water source

Left absolutely more important (9)	Left much more important (7)	Left comparatively more important (5)	Left slightly more important (3)	Equally important (1)	Right slightly more important (3)	Right comparatively more important (5)	Right much more important (7)	Right absolutely more important (9)
☐	☐	☐	☐	☐	☐	☐	☐	☐

Secondary Factors under "Disturbance Condition"

Description of factors:

Factor	Description
Road disturbance	Represents the distribution intensity of the road network. A higher road density generally means more frequent traffic and a greater risk of disturbance to waterbirds.
Visitor-stay disturbance	Represents the distribution of locations where visitors remain within the study area and is used to reflect the intensity of human disturbance to waterbird habitat.

Question 11：Road disturbance VS Visitor-stay disturbance

Left absolutely more important (9)	Left much more important (7)	Left comparatively more important (5)	Left slightly more important (3)	Equally important (1)	Right slightly more important (3)	Right comparatively more important (5)	Right much more important (7)	Right absolutely more important (9)
☐	☐	☐	☐	☐	☐	☐	☐	☐

Secondary Factors under "Shelter / Cover"

Description of factors:

Factor	Description
Vegetation height	The vertical distance from the ground to the natural top of the vegetation. It determines vertical stratification within the habitat and is directly related to waterbirds' concealment requirements, nesting support, and ease of movement.
Slope	Represents terrain relief. Slope affects the accessibility of waterbird activities and concealment conditions; flatter areas are generally more favorable for waterbird activity.
Vegetation cover	The percentage of ground area covered by the vertical projection of the vegetation canopy, indicating the degree of horizontal concealment. It determines the balance between habitat openness and concealment

Factor	Description
	and affects waterbird safety and visibility while foraging.
Number of patches	Reflects the spatial distribution and connectivity of waterbird habitat. Changes in patch number affect habitat distribution patterns and relative isolation, potentially influencing waterbird roosting, migration, and foraging.

Question 12：Vegetation height VS Slope

Left absolutely more important (9)	Left much more important (7)	Left comparatively more important (5)	Left slightly more important (3)	Equally important (1)	Right slightly more important (3)	Right comparatively more important (5)	Right much more important (7)	Right absolutely more important (9)
☐	☐	☐	☐	☐	☐	☐	☐	☐

Question 13：Vegetation height VS Vegetation cover

Left absolutely more important (9)	Left much more important (7)	Left comparatively more important (5)	Left slightly more important (3)	Equally important (1)	Right slightly more important (3)	Right comparatively more important (5)	Right much more important (7)	Right absolutely more important (9)
☐	☐	☐	☐	☐	☐	☐	☐	☐

Question 14：Vegetation height VS Number of patches

Left absolutely more important (9)	Left much more important (7)	Left comparatively more important (5)	Left slightly more important (3)	Equally important (1)	Right slightly more important (3)	Right comparatively more important (5)	Right much more important (7)	Right absolutely more important (9)
☐	☐	☐	☐	☐	☐	☐	☐	☐

Question 15：Slope VS Vegetation cover

Left absolutely more important (9)	Left much more important (7)	Left comparatively more important (5)	Left slightly more important (3)	Equally important (1)	Right slightly more important (3)	Right comparatively more important (5)	Right much more important (7)	Right absolutely more important (9)
☐	☐	☐	☐	☐	☐	☐	☐	☐

Question 16：Slope VS Number of patches

Left absolutely more important (9)	Left much more important (7)	Left comparatively more important (5)	Left slightly more important (3)	Equally important (1)	Right slightly more important (3)	Right comparatively more important (5)	Right much more important (7)	Right absolutely more important (9)
☐	☐	☐	☐	☐	☐	☐	☐	☐

Question 17：Vegetation cover VS Number of patches

Left absolutely more important (9)	Left much more important (7)	Left comparatively more important (5)	Left slightly more important (3)	Equally important (1)	Right slightly more important (3)	Right comparatively more important (5)	Right much more important (7)	Right absolutely more important (9)
☐	☐	☐	☐	☐	☐	☐	☐	☐

Secondary Factors under “Food Abundance”

Description of factors:

Factor	Description
Animal-based food	Animal organisms obtained by waterbirds from aquatic or wetland environments through predation, filter feeding, picking/scavenging, and other feeding methods; these constitute an important source of nutrients for waterbirds.
Plant-based food	The coverage and biomass conditions of aquatic or wetland vegetation, which can serve as an indirect indicator of plant-food resources for swimming waterbirds and other waterbird groups.

Animal-based food: benthic animals and small aquatic animals, etc.; Plant-based food: aquatic plants and their seeds, rhizomes, etc.

Question 18：Animal-based food VS Plant-based food

Left absolutely more important (9)	Left much more important (7)	Left comparatively more important (5)	Left slightly more important (3)	Equally important (1)	Right slightly more important (3)	Right comparatively more important (5)	Right much more important (7)	Right absolutely more important (9)
☐	☐	☐	☐	☐	☐	☐	☐	☐

Please make sure that all pairwise comparisons have been completed to ensure the completeness and validity of the survey results.

Thank you for taking the time to participate in this questionnaire. The survey data will be used solely for scientific research. We sincerely appreciate your support!
